# Clinical significance and correlation of microRNA-21 expression and the neutrophil-lymphocyte ratio in patients with acute myocardial infarction

**DOI:** 10.6061/clinics/2019/e1237

**Published:** 2019-10-30

**Authors:** Changkui Gao, Dan Zhao, Jingjing Wang, Ping Liu, Baohe Xu

**Affiliations:** I Emergency Department, Daqing Longnan Hospital, Daqing, Heilongjiang, China; II Coronary Care Unit, Daqing Longnan Hospital, Daqing, Heilongjiang, China

**Keywords:** Acute Myocardial Infarction, miR-21, NLR, ROC

## Abstract

**OBJECTIVES:**

To explore the clinical significance and correlation of microRNA-21 (miR-21) and the neutrophil-lymphocyte ratio (NLR) in patients with acute myocardial infarction (AMI).

**METHODS:**

The observation group contained 184 patients, while the control group contained 150 patients. The expression of miR-21 in the serum of each group was detected by qRT-PCR.

**RESULTS:**

A total of 184 patients and their family members were followed-up for 30 days, among which 35 patients died and 149 patients survived, resulting in a survival rate of 80.97%. According to univariate analysis, there were significant differences in age, cardiac troponin (cTn), heart rate, Killip grade, percutaneous coronary intervention (PCI) operation rate, miR-21 and NLR. In the receiver operating characteristic (ROC) analysis, the area under the curve (AUC) values of miR-21 and NLR for the diagnosis of AMI were 0.909 and 0.868, respectively, and the area under the combined detection curve was 0.960. In the Kaplan-Meier survival analysis, the survival of patients with high miR-21 expression and NLR was significantly higher than that of patients with low miR-21 expression and NLR (*p*=0.027; *p*=0.001). The correlation showed that miR-21 expression in serum was positively correlated with the NLR in the observation group (r=0.528, *p*<0.05). cTn, heart rate, Killip classification, PCI operation rate, miR-21, NLR are independent risk factors for AMI.

**CONCLUSION:**

miR-21 and NLR play a role in the diagnosis of AMI and can be used as predictors for the survival of AMI.

## INTRODUCTION

Cardiovascular disease (CVD) is the most commonly occurring disease with the highest morbidity (1). The study by Go et al. (2) estimated that the mortality of CVD will increase to 36.6% worldwide by 2020, causing great harm to the lives and health of people. Other studies (3) indicated that there is a close relationship between CVD and atherosclerosis (AS). Patients with AS are prone to acute myocardial infarction (AMI), the main cause of which is coronary AS leading to stenosis of the vascular lumen, myocardial ischemia, hypoxia and myocardial damage. Boersma et al. (4) indicated that there were approximately 3 million new cases of patients with AMI worldwide every year. At present, cardiac troponin (cTn) is the main detection index for AMI and is recognized as the “gold diagnostic standard” (5). White et al. (6) demonstrated that the expression of cTn in the blood of patients with end-stage renal disease is elevated, which can be used as a biomarker of renal failure. Similarly, finding better biomarkers for the diagnosis of AMI is particularly important.

MicroRNAs (miRNAs) are a kind of noncoding short-chain RNA that are approximately 22 nucleotides in length. The main function of miRNAs is to inhibit the translation and transcription of target genes by binding to the 3’ untranslated region (UTR) of its downstream target gene mRNA, thus changing the expression of the target gene (7). Studies (8-10) have shown that miRNAs have regulatory effects in various diseases, such as tumors and cardiovascular diseases, as well as in altering neurological function. As one of the most widely studied miRNAs in recent years, miR-21 plays an important role in embryonic development, tumors, immune response and osteogenic differentiation (11). Dong et al. (12) showed that miR-21 was expected to be a potential diagnostic indicator of AMI instead of a predictor of death in AMI. In recent years, many studies have suggested that the neutrophil-lymphocyte ratio (NLR) is of great value in predicting coronary artery disease (13). Nilsson et al. (14) indicated that the higher the expression of the NLR is in non-ST-segment elevation myocardial infarction, the higher the rate of atheromatous plaque. Stakos et al. (15) demonstrated that the NLR could be used as an independent risk factor for thrombosis, in-stent restenosis, percutaneous transluminal coronary angioplasty (PTCA), coronary artery bypass graft, posterior coronary atherosclerotic plaque rupture and other CVDs.

Therefore, this study aimed to analyze the expression of miR-21 and NLR in patients with AMI to determine whether they can be used as indicators for the diagnosis and survival assessment of AMI, thereby providing a reference for the diagnosis and survival prediction of AMI.

## MATERIALS AND METHODS

### Clinical data of patients

A total of 184 patients with AMI treated at our hospital were allocated the observation group in this study, including 124 male patients and 60 female patients, aged 27 to 88 years, with a mean age of 59.0±9.4 years. In addition, 150 patients were allocated to the control group, including 102 male patients and 48 female patients, aged 29 to 89 years, with a mean age of 60.4±11.5 years. The study was approved by the medical ethics committee of the Daqing Longnan Hospital. All patients signed an informed consent form.

### Inclusion of exclusion criteria

The inclusion criteria were as follows. Patients were admitted to the hospital within 6 hours. The diagnostic criteria of AMI were based on the diagnostic guidelines for AMI issued by the American Heart Association (AHA) in 2003 (16). The marker for diagnosis of myocardial necrosis significantly increased. Patients with complete clinical data accepted the treatment and follow-up study.

The exclusion criteria were as follows: patients with congenital immunodeficiency, acute infectious diseases, valvular heart diseases, malignant tumors, liver and kidney dysfunction, a history of severe bleeding, severe bleeding, and antiplatelet aggregation therapy.

### Main kits and instruments

A hematology analyzer (Siemens, Germany, ADVIA2120i), a PCR system (ABI Company, USA, 7500), a Total RNA Extraction Kit, an EasyPure miRNA Kit, a Reverse transcription+PCR Kit, and the TransScript miRNA First-Strand cDNA Synthesis SuperMix (TransGen Biotech, Beijing, China, ER601-01, AT351-01) were used. The miR-21 primers were synthesized by Shanghai Sangon Biotech Co., Ltd. (Table 1).

## Methods

### Patients after treatment

Patients diagnosed with AMI were treated with 300 mg aspirin +300 mg clopidogrel or 180 mg ticagrelor.

### Specimen collection

Eight milliliters of fasting venous blood was collected from patients who received a physical examination. Eight milliliters of abdominal venous blood was collected from patients in the observation group. The collected blood samples were placed in EDTA anticoagulant tubes (3 mL) and inert separation gels and coagulant tubes (5 mL). The expression of NLR in venous blood in the EDTA anticoagulant tubes was detected by flow cytometry. The venous blood in inert separation gels and coagulant tubes was centrifuged at 3000 rpm and 24°C for 10 min to collect the serum for the next step, and the remaining sample was placed in an EP tube without RNA enzymes at -80°C.

### PCR detection

An EasyPure miRNA Kit was used to extract total RNA. Total RNA was extracted by an ultraviolet spectrophotometer, and agarose gel electrophoresis (AGE) was used to determine the purity, concentration and integrity of the total RNA. TransScript^®^ miRNA RT Enzyme Mix and 2×TS miRNA Reaction Mix were used to reverse transcribe the total RNA according to the manufacturer’s instructions. PCR amplification experiments were carried out. The PCR system was as follows: 1 μL of cDNA, 0.4 μL of upstream and downstream primers, 10 μL of 2×TransTaq^®^ Tip Green qPCR SuperMix, 0.4 μL of Passive Reference Dye (50×) and 20 μL of ddH2O. The PCR conditions were as follows: predenaturation at 94°C for 30s, denaturation at 94°C for 5s, annealing at 60°C for 30s, with a total of 40 cycles. Three replicate wells were set for each sample, and the experiment was performed three times. In this study, U6 was used as an internal reference, and the 2^-△ct^ method was used to analyze the data.

### Observation index

The main outcome measures were as follows. The expression levels of miR-21 and NLR were compared between the observation group and the control group. Survival at 30 days after admission was calculated. According to the survival of patients, the risk factors for death were analyzed by multivariate logistic regression. Pearson’s test was used to analyze the correlation between miR-21 and NLR in the serum of the observation group.

The secondary outcome measures were as follows. Receiver operating characteristic (ROC) curves were used to analyze the diagnostic values and best cut-off values of miR-21 and NLR in patients with AMI. The best cut-off values of miR-21 and NLR were used to divide patients into high- and low-expression groups to observe the survival of patients in 30 days and plot the Kaplan-Meier (K-M) curve.

### Statistical analysis

In this study, the SPSS 20.0 (Shanghai Cabit Information Technology Co., Ltd., China) software package was used to analyze the collected data. GraphPad Prism 7 (Shenzhen Softhead Software Technology Co., Ltd., China) was used to plot the data. The count data were assessed by chi-square test (χ^2^). The Kolmogorov-Smirnov (K-S) test was used to analyze the distribution of the data. The measurement data are expressed as the means ± standard deviations (SDs). The normally distributed data were compared by independent sample t test. The rank data were assessed by the rank sum test. The nonparametric test was used for the nonnormally distributed data. Pearson’s test was used to analyze the relationship between miR-21 and NLR in the serum of the observation group. The ability of miR-21 and NLR to diagnose AMI was evaluated by ROC analysis. K-M survival curves and the log rank test were used to analyze the 30-day survival of patients. *p*<0.05 indicated a statistically significant difference.

## RESULTS

### Clinical data of the patients

According to the clinical data, there were 124 male patients and 60 female patients in the observation group, with an average age of 59.0±9.4 years and an average BMI of 23.15 kg/m^2^. Among these patients, 55 had hypertension, 12 had diabetes, 24 had hyperlipidemia, 16 had chronic obstructive pulmonary disease (COPD), 130 had a history of smoking, and 20 had a history of alcoholism. A total of 152 patients were urban residents, and 32 were rural residents. There were 102 male patients and 48 female patients in the control group, with an average age of 60.4±11.5 years and an average BMI of 23.04 kg/m^2^. Among these patients, 39 had hypertension, 14 had diabetes, 23 had hyperlipidemia, 10 had COPD, 115 had a history of smoking, and 11 had a history of alcoholism. A total of 130 patients were urban residents, and 20 were rural residents. There was no significant difference between the two groups (*p*>0.05). There was no difference in total cholesterol, triglyceride, low-density lipoprotein, creatinine or urea nitrogen concentration between the two groups (*p*>0.05). The concentration of cTn in the observation group was significantly higher than that in the control group (*p*<0.05) (Table 2).

### Expression of miR-21 and NLR in the observation group and control group

**Figure 1:**
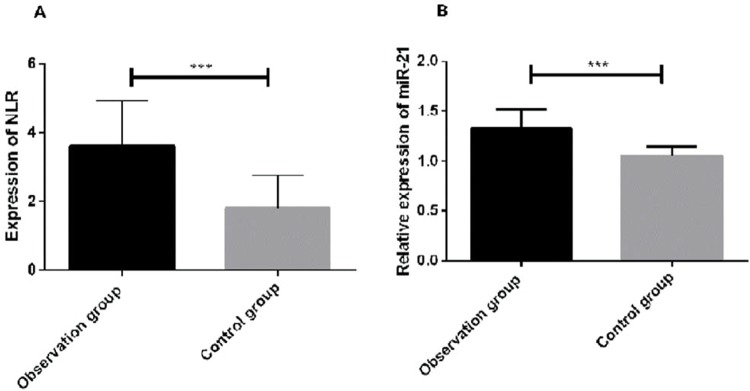
Expression of miR-21 and the NLR in the observation group and the control group. The NLR in the peripheral blood of the patients in the observation group was significantly higher than that in the patients in the control group (*p*<0.05). The expression of miR-21 in the serum of the patients in the observation group was significantly higher than that of the patients in the control group (*p*<0.05). *The expression of miR-21 was significantly higher in the observation group than in the control group (*p<*0.001).

Regarding the expression of miR-21 and NLR in the two groups, the expression of miR-21 and NLR in the observation group was significantly higher than that in the control group (*p*<0.05) (Figure 1).

### Diagnostic value of miR-21 and NLR in AMI

**Figure 2:**
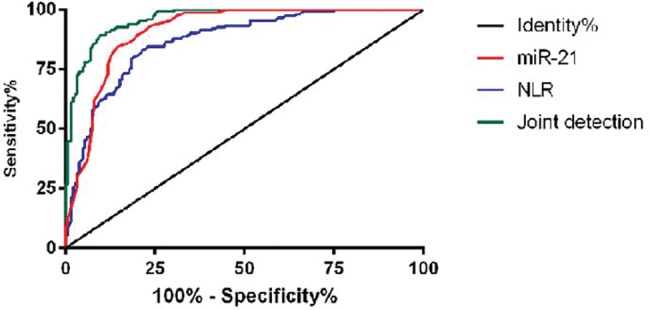
ROC curve of miR-21 and NLR for the diagnosis of AMI.

ROC curves were plotted to analyze the diagnostic value of miR-21 and NLR in AMI between the observation group and the control group. The area under the curve (AUC) of miR-21 was 0.909 (95% CI: 0.877∼0.941). The AUC of NLR was 0.868 (95% CI: 0.830∼0.906). The AUC of the combined detection curve was 0.960 (95% CI: 0.943∼0.978) (Figure 2 and Table 3).

### Survival of patients

**Figure 3:**
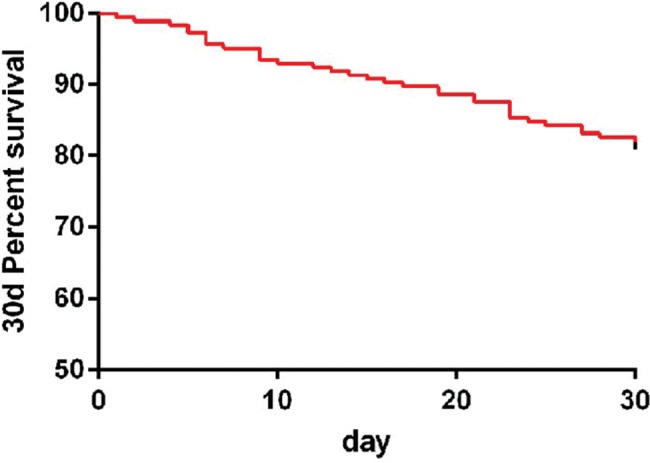
The survival of the patients was followed up for 30 days. The total survival rate at 30 days was 80.97.

For the 30-day survival of the patients in the observation group, 184 patients were followed-up, among which 35 patients died within 30 days and 149 patients survived, resulting in a survival rate of 80.97% (Figure 3).

### Diagnostic value of miR-21 and NLR for AMI

**Figure 4:**
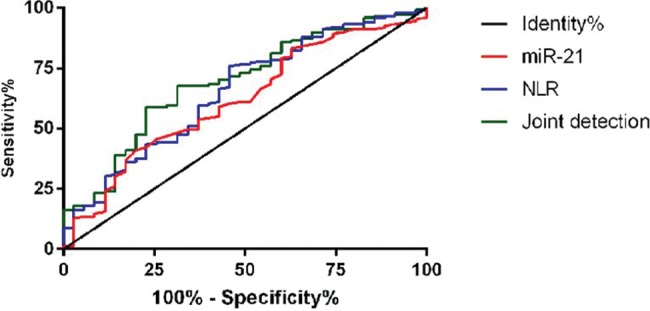
ROC curve of miR-21 and NLR for the diagnosis of AMI death.

ROC curves were used to analyze miR-21 and NLR in the diagnosis of AMI according to the survival and death of patients. The AUC of miR-21 was 0.617 (95% CI: 0.517-0.719). The AUC of NLR was 0.654 (95% CI: 0.552∼0.755). The AUC of the combined detection curve was 0.692 (95% CI: 0.597∼0.787) (Figure 4 and Table 4).

### Relationship between miR-21 expression and the NLR and the 30-day survival of patients

**Figure 5:**
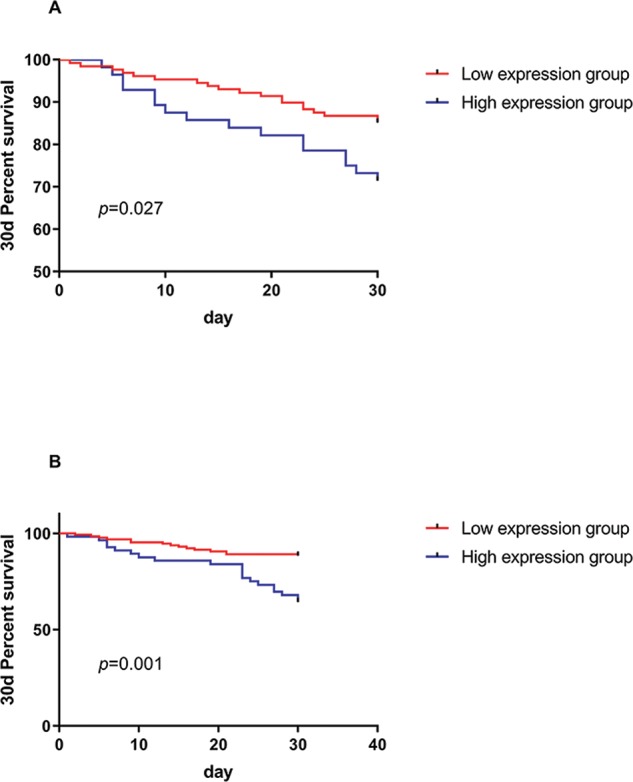
The relationship between the expression of miR-21 and NLR and the survival of patients at 30 days. A. The survival rate of patients was found to be significantly lower in the miR-21 high-expression group than in the low-expression group, and there was a significant difference between the high-expression group and the low-expression group. B. The survival rate of the NLR high-expression group was significantly lower than that of the low-expression group. There was a significant difference in 30-day survival.

High and low-expression groups were established according to the cut-off value of the ROC curve of miR-21 and NLR for the survival of AMI patients. Based on the K-M survival curve, the 30-day survival of patients with high miR-21 expression was significantly lower than that of patients with low miR-21 expression (*p*=0.027). Based on the NLR of the two groups, the 30-day survival of patients with a high NLR was significantly lower than that of patients with a low NLR (*p*=0.001) (Figure 5).

### Univariate analysis of patient survival

According to the survival status of the two groups, patients were divided into the survival group (n=149) and the death group (n=35). The univariate analysis based on the clinical data showed that there was no difference in sex, BMI, past medical history, smoking history, history of alcoholism, place of residence, total cholesterol, triglyceride, low-density lipoprotein, creatinine and urea nitrogen between the two groups (*p*>0.05). However, there were significant differences in age, cTn, heart rate, Killip classification, percutaneous coronary intervention (PCI) operation rate, miR-21 and NLR (*p*<0.05) (Table 5).

### Multivariate analysis of patient survival

The index of the variables with significant differences in the univariate analysis are shown in Table 6. Then, multivariate logistic regression analysis was performed. The results showed that age was not an independent risk factor for the survival of patients. However, cTn (OR: 6.435, 95% CI: 1.342∼30.861), heart rate (OR: 1.072, 95% CI: 1.033∼1.111), Killip classification (OR: 6.784, 95% CI: 2.265∼20.324), PCI operation rate (OR: 0.309, 95% CI: 0.117∼0.819), miR-21 (OR: 0.315, 95% CI: 0.118∼0.835), and NLR (OR: 0.310, 95% CI: 0.120∼0.799) were independent risk factors in patients with AMI (Table 7).

### Correlation between miR-21 and NLR in the observation group

**Figure 6:**
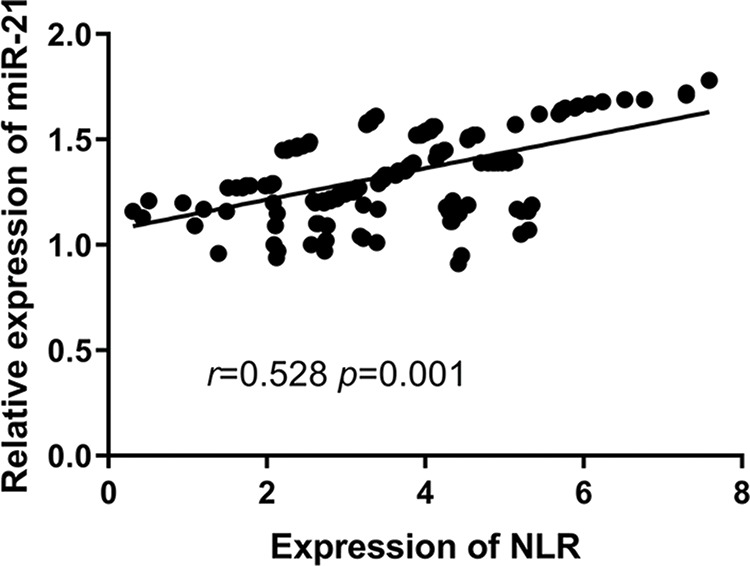
Positive correlation between miR-21 expression and NLR (*r*=0.528, *p*=0.001).

Regarding the relationship between miR-21 and NLR, miR-21 expression was positively correlated with NLR in the observation group; that is, the higher the NLR is, the higher expression of miR-21 (*r*=0.528, *p*=0.001) (Figure 6).

## DISCUSSION

As the most common clinical disease, AMI is characterized by a high incidence, high hospitalization rate and high mortality (17). Patients suffer from AMI because of the imbalance between the supply and demand of the myocardial cell energy supply, which leads to myocardial cell necrosis, or even cardiac dysfunction (18). At present, early diagnosis is the main treatment for patients with AMI. Revascularization can improve the condition of patients. Thus, the early diagnosis of AMI is particularly important. Currently, the diagnosis of AMI includes the observation of vital signs, imaging examination and traditional hematological examination. The presence of chest pain is an important feature for diagnosing AMI (19). However, traditional hematological tests, such as cTnI and CK-MB, have time limitations. In the early stage, the test results are negative and are determined by repeated measurements, which not only delay treatment but also increase the economic burden of patients (20).

As a hot topic of research in recent years, miRNAs have attracted the attention of many scholars. miRNAs are small noncoding RNAs approximately 21-25 nucleotides in length and are involved in cellular and physiological processes, such as the regulation of cells, energy metabolism and apoptosis. When a miRNA is bound to the 3’UTR of the downstream target gene, the mRNA is degraded, and the translation of the protein is inhibited (21). Many studies have indicated that multiple miRNAs are differentially expressed during the development of AMI (22,23). Specifically, miR-21 is an important miRNA. Previous studies have shown that miR-21 is differentially expressed between the serum of patients with AMI and that of normal people. Other studies have shown that miR-21 plays an important role in a variety of CVDs (24). Oerlemans et al. (25) indicated that the AUC of miR-21 combined with high-sensitivity troponin T for the diagnosis of acute coronary syndrome was found to be more than 0.9 by detecting the expression of miR-21 in 332 suspected acute coronary syndrome patients. However, few studies have shown that miR-21 can be used as a predictor of death in AMI. In this study, by detecting the expression of miR-21 in the plasma of the observation group and the control group, the expression of miR-21 in the serum of the observation group was found to be significantly increased. Zhang et al. (26) showed that the overexpression of miR-21 in serum can be used as a diagnostic indicator of acute ischemic heart disease. In addition, there was a difference in the expression of miR-21 between patients with AMI and normal patients, suggesting that miR-21 may be a potential diagnostic indicator of AMI. According to the ROC curve, the AUC of miR-21 was 0.909, indicating that miR-21 can be used as a biomarker of AMI. The NLR is an effective and inexpensive diagnostic indicator of inflammation. Some studies have shown that the NLR is not only involved in the process of AS but is also closely related to clinical events, such as atherosclerotic plaque collapse (27). In recent years, studies have demonstrated that the NLR has an outstanding effect on the diagnosis of AMI and can be used as a prognostic indicator for coronary heart disease (28,29). Therefore, this study detected the NLR in the plasma of patients with AMI and found that the NLR in the observation group was significantly higher than that in the control group. Aya et al. (30) suggested that the NLR in the blood of patients with ST-segment elevation myocardial infarction was significantly increased, which was consistent with our findings. According to the ROC curve, the AUC of NLR was 0.868, suggesting that the NLR has a certain value in the diagnosis of AMI. Based on the combined ROC curve of NLR and miR-21, the AUC was 0.960. When the cut-off value was 0.500 or below, the specificity and sensitivity were 90.23% and 89.33%, respectively, which were significantly higher than that of the single detections.

Furthermore, a follow-up study was conducted on the 30-day survival of the patients in the observation group. A total of 184 patients were included the follow-up study, of which 35 patients died and 149 patients survived within 30 days, with a survival rate of 80.97%. The patients were divided according to their survival status, and the diagnostic value of the NLR and miR-21 expression in patients who died from AMI was predicted by the ROC curve. The AUC of miR-21 was 0.617. The two indicators showed that the cut-off value was 1.265 or below, and the specificity and sensitivity were 80.00% and 40.94%, respectively. The AUC of NLR was 0.654. When the cut-off value was 4.245, the specificity and sensitivity were 54.29% and 75.84%, respectively. Given that differences existed in the sensitivity and specificity of these two indices, the AUC of the combined detection was 0.692. When the cut-off value was 0.208 or below, the specificity and sensitivity were 68.77% and 67.78%, respectively, which indicated that the combined detection could complement the defects. Moreover, patients were divided into high and low-expression groups according to the cut-off value. According to the K-M survival curve, the 30-day survival of patients with high NLR and miR-21 expression was significantly lower than that of patients with low NLR and miR-21 expression. According to multivariate logistic regression analysis, the low expression of miR-21 and NLR were protective factors for death of patients, indicating that the expression of miR-21 and NLR in patients can be used as a predictor of 30-day survival in patients with AMI.

Furthermore, Pearson’s test was used to detect the correlation between miR-21 and NLR in the observation group. The expression of miR-21 was positively correlated with the NLR, suggesting that a close relationship existed between miR-21 and the NLR. However, this study still has some limitations. First, the detection of miR-21 is more expensive than that of NLR. Long-term detection increases the economic burden of patients. Second, the relationship between miR-21 and NLR in clinical experiments is unclear. Relevant experiments in future research will be added to explore the relationship between miR-21 and NLR and prove the results of the research.

## CONCLUSIONS

In summary, cTn, heart rate, Killip classification, PCI operation rate, miR-21 and NLR are independent factors for death in patients with AMI. There is a positive correlation between miR-21 and NLR, which can be used as a predictor of the diagnosis and death of patients with AMI.

## AUTHOR CONTRIBUTIONS

Gao C was responsible for the study design and planning. Zhao D was responsible for the data collection and entry. Wang J was responsible for the data analysis and statistics. Gao C and Xu B were responsible for the data interpretation. Liu P was responsible for the manuscript preparation. Xu B was responsible for the literature analysis and search.

## Figures and Tables

**Table 1:** Primer sequences.

Gene	Upstream primer	Downstream primer
miR-21	5’-ACACTCCAGCTGGGTAGCTTATCAGACTGAT-3’	5’-ACTGGTGTCGTGGAGTCG-3’
U6	5’-CTCGCTTCGGCAGCACA-3’	5’-AACGCTTCACGAATTTGCGT-3’

**Table 2:** Clinical data of patients.

Factor		Observation group (n=184)	Control group (n=150)	t/2/Z value	*p-*value
Sex	Male	124 (67.39)	102 (68.00)	0.014	0.906
	Female	60 (32.61)	48 (32.00)		
Age (years)		59.0±9.4	60.4±11.5	1.224	0.222
BMI (kg/m^2^)		23.15±1.82	23.04±1.97	0.529	0.567
Anamnesis					
Hypertension		55 (29.89)	39 (41.49)	0.619	0.432
Diabetes mellitus		12 (6.52)	14 (9.33)	0.910	0.340
Hyperlipemia		24 (13.04)	23 (15.33)	0.358	0.549
COPD		16 (8.70)	10 (6.67)	0.474	0.491
Smoking history	Yes	130 (70.65)	115 (76.67)	1.529	0.216
	No	54 (29.35)	35 (23.33)		
History of alcoholism	Yes	20 (10.87)	11 (7.33)	1.227	0.268
	No	164 (89.13)	139 (92.67)		
Domicile	City	152 (82.61)	130 (86.67)	1.035	0.309
	Village	32 (17.39)	20 (13.33)		
Total cholesterol (mmol/L)		4.45±1.10	4.23±0.94	1.939	0.053
Glycerin trilaurate (mmol/L)		1.91±0.93	1.84±0.0.7	0.762	0.446
LDL (mmol/L)		2.56±0.87	2.44±0.76	1.326	0.186
Creatinine (μmol/L)		71.44±11.90	70.18±11.02	0.995	0.320
Urea nitrogen (mmol/L)		6.24±1.52	6.15±1.06	0.614	0.540
cTn (ng/mL)		0.69±0.35	0.09±0.06		<0.001

Note: COPD: chronic obstructive pulmonary disease

**Table 3:** ROC curve data.

Index	AUC	95% CI	Specificity	Sensitivity	Youden index	Cut-off
miR-21	0.909	0.877∼0.941	85.33%	84.66%	69.99%	<1.165
NLR	0.868	0.830∼0.906	77.17%	84.66%	61.84%	<3.035
Joint detection	0.960	0.943∼0.978	90.23%	89.33%	79.55%	<0.500

Note: AUC: area under the curve; Cut-off: intercept

**Table 4:** Diagnostic value of miR-21 and NLR in AMI death.

Index	AUC	95% CI	Specificity	Sensitivity	Youden index	Cut-off
miR-21	0.617	0.517∼0.719	80.00%	40.94%	20.94%	<1.265
NLR	0.654	0.552∼0.755	54.29%	75.84%	30.12%	<4.245
Joint detection	0.692	0.597∼0.787	68.77%	67.78%	36.36%	<0.208

Note: AUC: area under the curve; Cut-off: intercept

**Table 5:** Single-factor analysis.

Factor		Survival group (n=149)	Death group (n=35)	t/2/Z value	*p*-value
Sex	Male	99 (66.44)	25 (71.43)	0.320	0.571
	Female	50 (33.56)	10 (28.57)		
Age (years)	≥60	59 (39.60)	24 (68.57)	9.609	0.002
	<60	90 (60.40)	11 (31.43)		
BMI (kg/m^2^)		23.55±1.34	23.06±1.90	1.786	0.076
Anamnesis					
Hypertension		44 (28.76)	11 (27.50)	0.025	0.875
Diabetes mellitus		9 (6.04)	3 (8.57)	0.298	0.585
Hyperlipemia		19 (12.75)	5 (14.29)	0.059	0.808
COPD		12 (9.40)	4 (11.43)	0.133	0.716
Smoking history	Yes	103 (69.13)	27 (77.14)	0.878	0.349
	No	46 (30.87)	8 (22.86)		
History of alcoholism	Yes	16 (10.74)	4 (11.43)	0.014	0.906
	No	133 (89.26)	31 (88.57)		
Domicile	City	122 (81.88)	30 (85.71)	0.290	0.590
	Village	27 (18.12)	5 (14.29)		
Total cholesterol (mmol/L)		4.62±1.11	4.42±1.10	0.961	0.338
Glycerin trilaurate (mmol/L)		2.03±0.87	1.87±0.94	0.964	0.336
LDL (mmol/L)		2.36±0.79	2.60±0.88	1.582	0.115
Creatinine (μmol/L)		69.42±11.82	71.91±11.90	1.12	0.264
Urea nitrogen (mmol/L)		6.41±1.72	6.19±1.47	0.699	0.486
cTn (ng/mL)		0.66±0.34	0.83±0.34	2.662	0.009
Heart rate (?/min)		82.45±13.32	94.66±15.82	4.703	<0.001
Killip classification	≥Level 3	16 (10.74)	15 (42.86)	20.871	<0.001
	<Level 3	133 (89.26)	20 (57.14)		
PCI operation rate	Yes	92 (61.74)	15 (42.86)	4.155	0.042
	No	57 (38.26)	20 (57.14)		
miR-21	<1.265	109 (73.15)	19 (54.29)	4.766	0.003
	≥1.265	40 (26.85)	16 (45.71)		
NLR	< 4.245	114 (76.51)	15 (42.85)	15.32	<0.001
	≥4.245	35 (23.49)	20 (57.14)		

**Table 6:** Assignment table.

Factor	Assignment
Age	≥60 years = 1, <60 years =0
cTn	Data are continuous variables using raw data analysis
Heart rate	Data are continuous variables using raw data analysis
Killip classification	≥Level 3 =1, < Level 3 =0
PCI operation rate	Yes=1, No=0
miR-21	<1.435=1, ≥1.435=0
NLR	<4.245=1, ≥4.245=0
Death status	Death=1, Survival =0

**Table 7:** Multivariate analysis of survival.

Factor	B	S.E.	Wals	Sig.	Exp (B)	Exp(B)	95% CI
						Lower limit	Upper limit
cTc	1.862	0.800	5.418	0.020	6.435	1.342	30.861
NLR	-1.171	0.483	5.876	0.015	0.310	0.120	0.799
miR-21	-1.156	0.498	5.387	0.020	0.315	0.118	0.835
Heart rate	0.069	0.019	13.858	0.000	1.072	1.033	1.111
Killip classification	1.915	0.56	11.697	0.001	6.784	2.265	20.324
PCI operation rate	-1.173	0.497	5.575	0.018	0.309	0.117	0.819
